# Early detection of occupational cholangiocarcinoma in a high-risk patient under intensive surveillance: a case study

**DOI:** 10.1186/s40792-024-01871-4

**Published:** 2024-03-22

**Authors:** Naoko Kounami, Sakae Maeda, Akihiro Kitagawa, Hideo Tomihara, Yuki Ushimaru, Nobuyoshi Ohara, Tomohira Takeoka, Mitsunobu Imasato, Ryohei Kawabata, Shingo Noura, Yumiko Yasuhara, Atsushi Miyamoto

**Affiliations:** 1 Department of Surgery, Sakai City Medical Center, 1-1-1 Ebarazi-Cho, Nishi-Ku, Sakai, Osaka Japan; 2 Department of Pathology, Sakai City Medical Center, 1-1-1, Ebarazi-Cho, Nishi-Ku, Sakai, Osaka Japan

**Keywords:** Occupational cholangiocarcinoma, Organic solvents' exposure, Hepatectomy

## Abstract

**Background:**

Occupational cholangiocarcinoma is associated with exposure to organic solvents, such as dichloromethane (DCM) and 1,2-dichloropropane (DCP). This report describes a case of occupational cholangiocarcinoma detected through regularly imaging following the discovery of elevated serum γ-glutamyl trans peptidase (γ-GTP) levels revealed during regular checkup.

**Case presentation:**

A 43-year-old man who had been working in a printing company with 15 years of exposure to organic solvents presented to our hospital owing to abnormalities found during a routine checkup. Ultrasound (US) imaging revealed thickening of the gallbladder wall accompanied by gallstones, although in the blood tests, γ-GTP levels were within normal range. Given the high risk of cholangiocarcinoma development, the patient underwent regular monitoring with abdominal US and blood tests at a local doctor's office. At the age of 48, his serum γ-GTP level mildly elevated for the first time, prompting the initiation of semi-annual magnetic resonance cholangiopancreatography (MRCP). By the age of 50 years, dilation in B8 was detected, and one and a half years later, a tumor on the central side of the B8 dilation appeared. The patient was diagnosed with intrahepatic cholangiocarcinoma, which was treated with anterior sectionectomy. Pathological examination revealed an adenocarcinoma with a papillary glandular ductal structure at the root of the B8. In addition, biliary intraepithelial neoplasia (BilIN) and dysplasia have been identified around the tumor and periphery bile ducts and in noncancerous bile ducts. Postoperatively, the patient received 6 months of adjuvant chemotherapy with S-1monotherapy. Eight months after surgery, the patient remained under observation with no signs of recurrence.

**Conclusions:**

We report a case of occupational cholangiocarcinoma detected during a prolonged period of regular follow-up after exposure to DCM and DCP. Given the delayed carcinogenesis process, occupational cholangiocarcinomas manifest long after exposure to organic solvents, therefore, ongoing screening is extremely important. Vigilance is essential to avoid underdiagnosis, particularly for individuals who are at an increased risk of developing this form of cancer. Continuous monitoring is key to the early detection and effective management of occupational cholangiocarcinoma.

## Background

Occupational cholangiocarcinoma was initially recognized as an occupational disease in 2013 by the Japanese Ministry of Health, Labor, and Welfare owing to an outbreak of cholangiocarcinoma among employees of a printing company in Osaka, Japan [1]. The cause was presumed to be long-term high-concentration exposure to organic solvents such as DCM and DCP. Furthermore, in 2014, the International Agency for Research on Cancer (IARC) classified DCP into Group 1 (carcinogenic to humans) and DCM into Group 2A (probably carcinogenic to humans) [2]. Exposure to high concentrations of DCM and/or DCP should raise concerns regarding late-onset carcinogenesis, even after exposure ceases [3]. Here, we report a case of occupational cholangiocarcinoma identified using serial imaging, triggered by abnormal laboratory values during a medical checkup, in a printing company employee.

## Case presentation

A 43-year-old man working at a printing company that had been exposed to organic solvents for 15 years (Table 1) was introduced to our hospital with thickening of the gallbladder wall observed on abdominal ultrasonography (US) during a routine medical checkup. He had no significant medical history, or history of smoking and alcohol consumption.

**Table 1:** History of exposure to organic solvents

Organic solvents	Exposure age	Duration of exposure
Dichloromethane	22–27 years old	About 4 years and 8 months
1,2-Dichloropropane	22–37 years old	About 15 years and 2 months

**Fig. 1:**
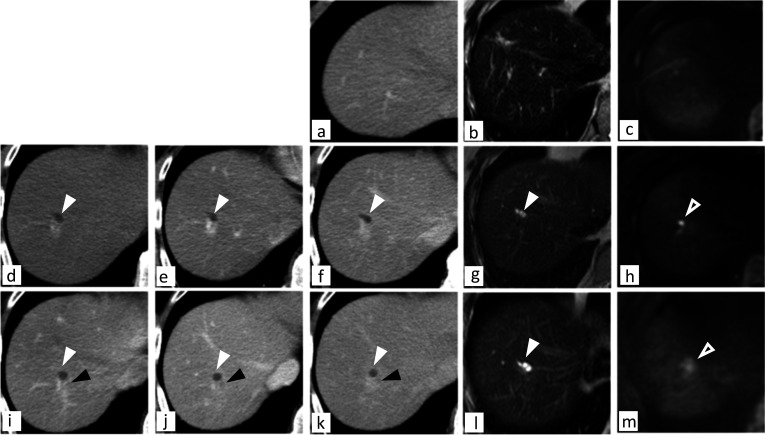
Temporal changes in abdominal CT and MRI findings. (**a**) Single-phase contrast-enhanced CT; (**d**, **i**) hepatic artery phase; (**e**, **j**) portal phase; and (**f**, **k**) equilibrium phase in dynamic abdominal CT imaging. T2-weighted (**b**, **g**, **l**) and DWI (**c**, **h**, **m**) MRCP images. Absence of abnormalities in the intrahepatic bile ducts during initial examination. (**a**, **b**, **c**) Images showing dilatation of B8 (**d**, **e**, **f**, **g**) and a small area of high signal intensity observed on DWI (**h**). Preoperative imaging revealing a tumor with ring enhancement at the root of B8 (**i**, **j**, **k**, **l**), corresponding to that area, and the high-signal intensity on DWI imaging increasing in size (**m**)

**Fig. 2:**
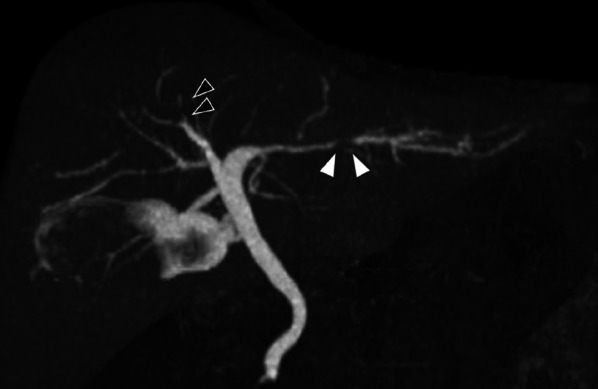
Preoperative DIC-CT findings. Black arrows: interruption and narrowing of the bile duct in the root area of segment B8. White arrows: narrowing of the bile duct in segment B2

Initial dynamic computed tomography (CT) of the abdomen revealed gallbladder wall thickening, suggesting chronic cholecystitis, with no lesions in the liver (Fig. 1). The patient continued to undergo regular abdominal US and blood tests at the local physician’s office because of the high risk of developing cholangiocarcinoma. At 47 years of age, the patient had a γGTP level of approximately 40 U/L. His regular checkup at 48 years revealed a mild elevation of the serum γGTP level which was 82 U/L and remained elevated between 80 and 100 U/L. Results of the laboratory tests performed when the patient was referred to our hospital revealed an elevated serum γGTP activity. However, the serum concentrations of aspartate aminotransferase (AST), alanine aminotransferase (ALT), total bilirubin (T-Bil), and carbohydrate antigen 19-9 (CA19-9) were within normal ranges (Table 2). Magnetic resonance cholangiopancreatography (MRCP) revealed thickening of the gallbladder wall and chronic cholecystitis without dilatation of the bile duct or presence of tumor in the hepatic or extrahepatic bile ducts (Fig. 1a–c). Dilatation of B8 was observed on dynamic CT and MRCP performed at 51 years of age. Diffusion-weighted imaging (DWI) revealed a small area of high-signal intensity corresponding to the center of the dilated bile duct in MRCP (Fig. 1g, h), whereas the dynamic CT was negative for any tumor lesions in the liver (Fig. 1d–f). Endoscopic retrograde cholangiopancreatography-guided (ERCP) brush cytology of B8 revealed no malignant findings. An MRCP repeated when he was 53-year old revealed further dilation of B8 (Fig. 1l) and enlargement of the high-signal intensity area on DWI at the root of B8 (Fig. 1m). Abdominal dynamic CT revealed a nodule with ring enhancement in the same area (Fig. 1i–k). Drip Infusion Cholecystocholangiography (DIC-CT) revealed stenosis of the roots of B8 and intermediate B2 (Fig. 2). No tumor lesions were identified using any imaging modality in the area with B2 stenosis. Based on these imaging findings, we suspected the development of malignancy and diagnosis of intrahepatic cholangiocarcinoma at roots of B8, stage T1aN0M0 according to the UICC 8th edition. Blood tests showed normal liver function, with a Child–Pugh score of 5 A and liver damage level A. There was no evidence of HBV or HCV infection. The tumor markers were within normal range with CEA at 1.6 ng/mL and CA19-9 antigen at 36.1 U/mL (Table 2).

**Table 2:** Trends in blood test results

Age	47	48	49	50	51	53
AST (U/L)	15	15	15	21	27	19
ALT (U/L)	16	14	17	27	44	23
ALP (U/L)	183	200	220	195	256	244
γ-GTP (U/L)	47	82	101	97	99	93
T-Bil (mg/dL)	0.6	0.8	0.9	0.7	0.58	0.74
CA19-9 (U/mL)	4	5	5	3	5	36.1
CEA (ng/mL)					1.2	1.6

*AST* aspartate aminotransferase, *ALT* alanine aminotransferase, *ALP* alkaline phosphatase, *γ-GTP* γ-glutamyl transpeptidase, *T-Bil* total bilirubin, *CA19-9* carbohydrate antigen 19-9, *CEA* carcinoembryonic antigen

In consideration of the stenosis in B2 and given the high likelihood of occupational cholangiocarcinoma in B8, based on the history of exposure to organic solvents, we assessed that there was a high risk of intrahepatic cholangiocarcinoma developing in the B2 region in the future. Consequently, we performed right anterior sectionectomy to preserve the remaining liver volume, rather than right hepatectomy, to ensure sufficient distance from the tumor. The operation time was 502 min, and the blood loss was 1175 ml without any transfusion. The patient was discharged on the 7th postoperative day without any complications. After surgery, postoperative adjuvant chemotherapy with S-1 monotherapy was administered for 6 months. The patient is currently on regular follow-up every 2–3 months with performance of blood tests including γGTP, CEA and CA19-9 and MDCT alternating with MRI and sometimes additional abdominal US. Moreover, 2 months postoperatively, the γ-GTP value decreased and remained within normal levels thereafter. Eight months postoperatively, the patient presented no signs of recurrence.

## Pathological findings

### Macroscopic findings

**Fig. 3:**
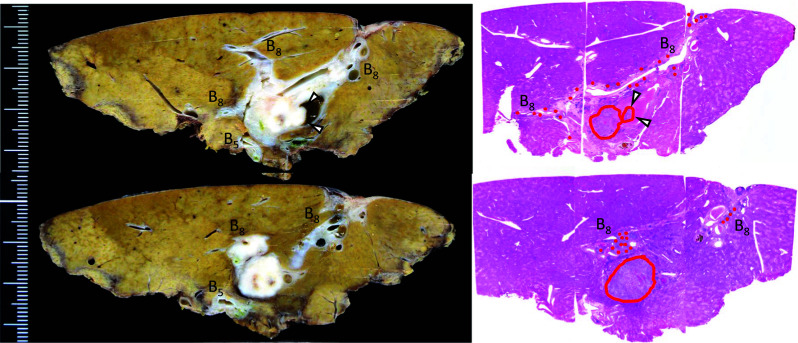
Macroscopic images of pathological findings. Red circle: Invasive carcinoma. Red dots: BilIN. White arrow: Protrusion of tumor in dilated bile duct in B8

A well-demarcated solitary tumor extending to the dilated bile ducts was found in the root of B8. The cut surface of the tumor was whitish with irregular, well-defined borders, suggestive of mass-forming cholangiocarcinoma. The bile duct walls in B8 peripheral to the tumor were dilated and thickened, with tethering observed on the liver surface (Fig. 3).

### Microscopic findings

**Fig. 4:**
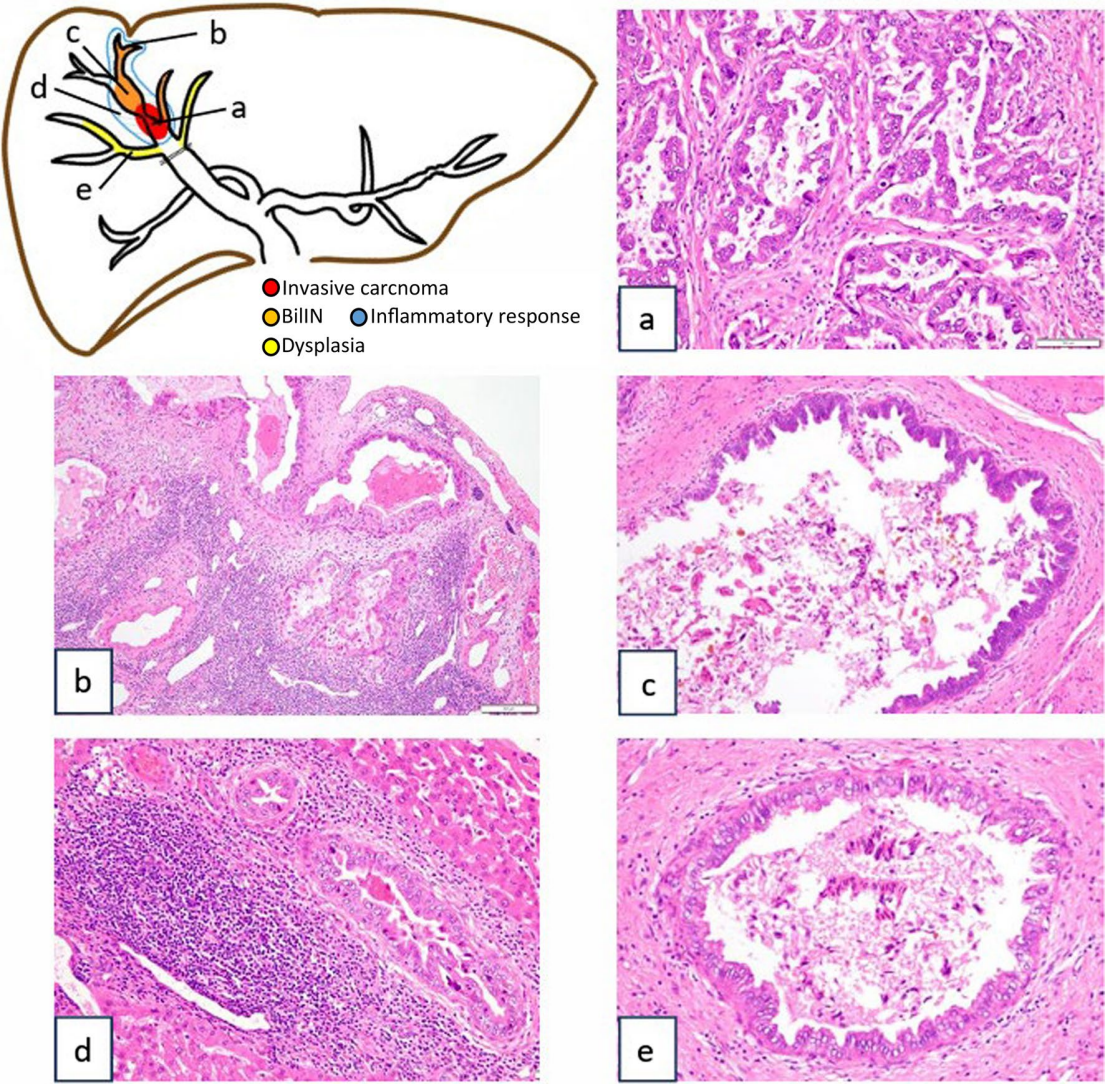
Mapping chart of pathological findings. **a** Adenocarcinoma with papillary glandular ductal structures at the root of B8. **b** Tethering on the liver surface owing to inflammation around bile ducts with BilIN. **c** BilIN at the peripheral side of tumor in B8. **d** Strong inflammatory response with lymphocytic infiltration around the main tumor and BilIN. **e** Dysplasia in B5

**Fig. 5:**
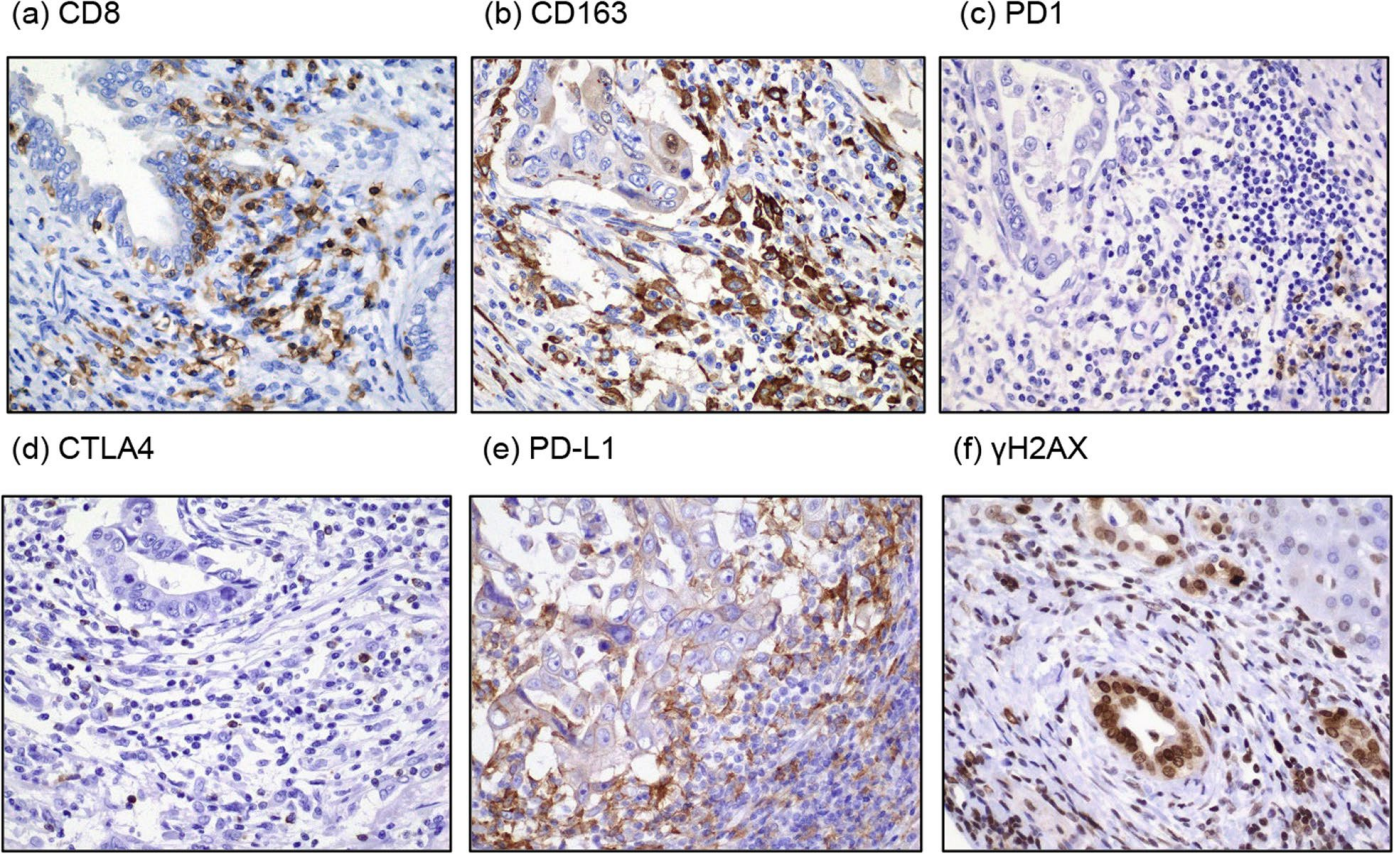
Pathological findings identified with immunological staining. **a** CD8-positive T cell infiltration in the tumor stroma. **b** CD163-positive macrophage infiltration in the tumor stroma. **c** PD1-positive lymphocytes in tumor stroma. **d** CTLA4-positive lymphocytes in the tumor stroma. **e** Enhanced PD-L1 expression in stromal cells of the invasive carcinoma. f γH2AX expression detected at various levels in the bile ducts irrespective of cancerous and non-cancerous sites

In the root of B8, an adenocarcinoma with a papillary glandular ductal structure was observed (Fig. 4a). Immunohistochemistry showed positivity for S100P, a marker of malignant transformation, leading to the diagnosis of well-differentiated intrahepatic cholangiocarcinoma of large duct type. Biliary intraepithelial neoplasia (BilIN) with disruption of cell polarity was observed in the dilated peripheral B8 distal to the tumor (Fig. 4c), and dysplasia was noted in B5, which converged with the right anterior sectional bile duct central to the tumor (Fig. 4e). Notably, a strong inflammatory response with lymphocytic infiltration was observed around the main tumor and BilIN in B8 (Fig. 4b, d), but not around the dysplasia in B5 (Fig. 4e). Immunohistochemistry revealed infiltration of CD8-positive T cells and CD163-positive macrophages in the tumor stroma (Fig. 5a, b). A relatively large number of PD1-positive lymphocytes was observed in the tumor stroma (Fig. 5c), whereas fewer CTLA4-positive lymphocytes were distributed in an interspersed manner (Fig. 5d). Programmed death-1 ligand (PD-L1) expression was abundant in the tumor stroma in the advanced infiltrative zone, and the PD-L1-positive cells were mainly macrophages (Fig. 5e). The combined positive score (CPS), defined as the number of PD-L1-staining cells (tumor cells, lymphocytes, and macrophages) divided by the total number of viable tumor cells multiplied by 100, was calculated at 64%. PD-L1 expression was detected in few cancer cells, and the rate of PD-L1 positivity in cancer cells was low. The majority of cells of the invasive carcinoma, BilIN, and non-cancerous bile ducts showed positive expression of γH2AX (Fig. 5f), which is a marker of double-stranded DNA injury.

## Discussion

The imaging findings of occupational cholangiocarcinoma consist in a localized intrahepatic bile duct dilatation without tumor formation [5]. In this case, intrahepatic bile duct dilatation appeared first, followed by tumor shadow, consistent with previous reports [2, 4, 5]. Prior to the detection of the mass lesion, MRI revealed a small area of hyperintensity on DWI. This hyperintensity was a diagnostic challenge, as it could suggest either obstructive cholangitis or neoplasia. ERCP was performed, however without providing definitive diagnosis of malignancy. Positron emission tomography-computed tomography (PET-CT) is an informative modality in evaluating tumor progression in occupational cholangiocarcinoma [6]. Based on this report, prior fluorodeoxyglucose (FDG) uptake assessment might had informed the surgical treatment decision-making process in our case.

Pathological examination revealed diffuse chronic bile duct damage, precancerous lesions, early-stage cancer, and cholangiocarcinoma. As previously reported, cancer follows a multistage carcinogenesis pattern, starting from chronic inflammation of the bile ducts and accessory gland epithelium, to dysplasia, precancerous lesions, and invasive cancer [7]. Immunopathological tests have detected γH2AX, a marker of double-stranded DNA damage, in various areas, including areas of bile ducts without tumor invasion, and the expression of S100P in BilIN, which indicate precancerous and early-stage cancer lesions [7]. DNA damage from past exposure to organic solvents induces multifocal and sporadic carcinogenesis and disease can manifest even long after exposure has ceased [3].

This patient was diagnosed with cholangiocarcinoma 16 years after the last exposure to DCM and DCP. Cholangiocarcinoma was identified at the root of segment B8, with the peripheral areas of B8 exhibiting BilIN changes. Conversely, segment B5, which remained unaffected by the tumor-induced obstruction, displayed dysplasia only. We hypothesized that all bile ducts within the rest of the liver would undergo changes similar to those observed in the B5. Consequently, similar alterations with presence of dysplasia would likely have been present in the roots and peripheral B8. The formation of a tumor at the root of B8 likely induces obstructive cholangitis, consequently leading to BilIN changes owing to inflammation. Given the widespread presence of dysplasia in B5, which indicates a significant risk of progression to cholangiocarcinoma in all remaining bile ducts, rigorous follow-up is paramount to detect recurrence early. Regarding the treatment strategy at the time of recurrence, occupational cholangiocarcinoma is known to have higher PD-L1 expression than ordinary cholangiocarcinoma [8]. One study reported a 100% complete response (CR) rate with nivolumab in a few patients with inoperable or recurrent occupational cholangiocarcinoma [9], suggesting its potential effectiveness in relapse setting. Restricting the surgical approach to anterior sectionectomy also provides flexibility for subsequent lateral sectionectomy, when de novo tumor emerges at the B2 constriction site, as indicated by the appearance of hyperintensity on DWI in MRI. When formulating a treatment strategy for occupational cholangiocarcinoma, it is crucial to consider the likelihood of recurrence and preserve a wide range of therapeutic options for effective future management.

## Conclusions

Here, we present a case of occupational cholangiocarcinoma in which early diagnosis and therapeutic intervention were possible through effective screening. Early detection of occupational cholangiocarcinoma in workers with a history of exposure to organic solvents may be provided through a combination of US and clinical tests including γ-GTP, AST, ALT, CA 19-9, and CEA [10]. In this case, only γ-GTP was elevated, whereas the tumor markers CEA and CA 19-9 did not increase. Careful follow-up with MRI enables a relatively early diagnosis and intervention. Considering the characteristic delayed carcinogenesis in occupational cholangiocarcinoma, which can occur long after exposure to organic solvents, continuous screening is extremely important to avoid underdiagnosis, particularly in patients at a risk of developing this disease.

## Data Availability

Data will be made available by the corresponding author upon request.

## Abbreviations

ALP: Alkaline phosphatase
ALT: Alanine aminotransferase
AST: Aspartate aminotransferase
BilIN: Biliary intraepithelial neoplasia
CA19-9: Carbohydrate antigen 19-9
CEA: Carcinoembryonic antigen
CPS: Combined positive score
CT: Computed tomography
DCM: Dichloromethane
DCP: Dichloropropane
DIC-CT: Drip infusion cholecystocholangiography
DWI: Diffusion-weighted imaging
ERCP: Endoscopic retrograde cholangiopancreatography
FDG: Fluorodeoxyglucose
γGTP: γ-Glutamyl trans peptidase
IARC: International Agency for Research on Cancer
MRCP: Magnetic Resonance Cholangiopancreatography
PET-CT: Positron emission tomography-computed tomography
PD-L1: Programmed death-1 ligand
T-Bil: Total bilirubin
US: Ultrasound
